# Identification of risk factors associated with disclosure of false positive bovine tuberculosis reactors using the gamma-interferon (IFNγ) assay

**DOI:** 10.1186/1297-9716-44-117

**Published:** 2013-12-05

**Authors:** Eamonn Gormley, Mairead Doyle, Anthony Duignan, Margaret Good, Simon J More, Tracy A Clegg

**Affiliations:** 1 Tuberculosis Diagnostics and Immunology Research Centre, School of Veterinary Medicine, University College Dublin, UCD, Belfield, Dublin 4, Ireland; 2 Department of Agriculture, Food & the Marine, Agriculture House, Kildare St, Dublin 2, Ireland; 3 Centre for Veterinary Epidemiology and Risk Analysis, UCD School of Veterinary Medicine, University College Dublin, Belfield, Dublin 4, Ireland

## Abstract

The gamma-interferon assay (IFNγ) is often used as an ancillary diagnostic test alongside the tuberculin skin test in order to detect *Mycobacterium bovis* infected cattle. The performance of the IFNγ test has been evaluated in many countries worldwide and wider usage as a disease surveillance tool is constrained due to the relatively low and inconsistent specificity at a herd and area level. This results in disclosure of a higher proportion of false positive reactors when compared with the skin test. In this study, we used cohorts of animals from low prevalence tuberculosis herds (*n* = 136) to assess a range of risk factors that might influence the specificity of the test. Univariate and multivariate logistic generalised estimating-equation (GEE) models were used to evaluate potential risk factors associated with a false positive IFNγ test result. In these herds, the univariate model revealed that the region of herd origin, the time of year when the testing was carried out, and the age of the animal were all significant risk factors. In the final multivariate models only animal age and region of herd origin were found to be significant risk factors. A high proportion of herds with multiple IFNγ false positive animals were located in one county, with evidence of within-herd clustering, suggesting a localised source of non-specific sensitization. Knowledge of the underlying factors influencing the IFNγ test specificity could be used to optimize the test performance in different disease level scenarios in order to reduce the disclosure rate of false positive reactors.

## Introduction

The detection of early *Mycobacterium bovis* (*M. bovis*) infection in cattle relies on the measurement of the cell-mediated immune response (CMI), which dominates in the early stages of tuberculosis and involves recruitment and activation of a variety of T cells to the site of infection [1]. These responses can be measured peripherally and this has lead to the development of several diagnostic assays that have proven effective in diagnosing tuberculosis in cattle [2]. The most widely used field surveillance test is the tuberculin skin test that measures the CMI response to *M. bovis* exposure [3]. The tuberculin used in cattle contains a crude mixture of predominantly secreted mycobacterial proteins derived from specified strains of *M. bovis *[4,5] and varies widely both in protein content and antigenic profile [6]. Thus, the differences in potency between various tuberculin products, defined as a measure of a tuberculin’s activity in animals sensitized with a specified organism, could be expected to influence the sensitivity and specificity of the skin test [7,8]. The reactive antigens are common to the members of the *M. tuberculosis* complex and tuberculin can be used to measure the CMI response as evidence of exposure to *M. bovis*. However, many of these antigens are also found in non-pathogenic environmental mycobacterial species and this cross reactivity to common antigens can result in a reduced specificity of the test, giving rise to non-specific reactors (false positives) [9,10]. Where this problem occurs, an *M. avium-derived* tuberculin is included to perform the single intradermal comparative tuberculin test (SICTT). Studies conducted in cattle populations free of bovine tuberculosis (TB) have shown that the specificity of the SICTT to be between 78.8% and 100% with median of 99.5% [2]. In a more recent study using latent class analysis (LCA) without a gold standard, the sensitivity and specificity was reported to be 52.9 - 60.6% and 99.2-99.8%, respectively [11]. Differences in performances are largely due to variation of testing techniques, including differences in tuberculin doses, tuberculin preparations, tuberculin potency and the interpretation of skin reactions [3].

Arising from the need to increase the detection rate of *M. bovis-*infected animals in exposed herds, the interferon-gamma assay (IFNγ) was developed as an ancillary test to improve the sensitivity of testing of cattle when used in parallel with the tuberculin skin test. The principle of the ELISA assay is to detect and quantify release of the IFNγ cytokine when heparinised whole blood is incubated with bovine and avian tuberculin (PPD), normally within the first 8-24 hours post-collection [12]. In a summary of many trials conducted to assess the performance characteristics of the IFN γ test, the sensitivity varied between 73.0% and 100%, with a median value of 87.6%. Its median specificity was 96.6%, with a range of 85.0–99.6% [2]. In initial studies conducted in Ireland the sensitivity and specificity of the IFN γ test were estimated at 56.2-87.7% and 88.1-96.6%, respectively, depending on the cut-off values used [10]. In a separate study, Gormley et al., estimated sensitivity to be 88% based on detection of tuberculous lesions at post-mortem and a specificity of 95% in herds with a 5-year TB free history [13]. LCA analysis on high and low TB prevalence herds in Ireland provided a sensitivity estimate of 63.1-70.1% and specificity of 86.8-89.4% [11]. Because its sensitivity appears to be higher than the SICTT, the IFNγ assay is primarily used to detect the maximum number of infected animals in a herd or in a region when interpreted in parallel with the tuberculin test. However, the increased sensitivity of the diagnostic regime can result in a decrease in diagnostic specificity. Both the source and concentration of tuberculins used in the IFNγ test has been shown to affect the performance of test [14]. The relatively low specificity of the IFNγ test in comparison with the SICTT has constrained its usage in TB free herds undergoing surveillance testing, as it is likely that an unacceptable number of false positive non-specific reactors would be identified.

To date there have been few reports of factors that may influence the specificity of the IFNγ test under natural conditions [15,16]. Non-specific reactions have been observed using the IFNγ assay in cattle in South Africa when the animals were exposed to the environmentally common, fast-growing and non-pathogenic *Mycobacterium fortuitum*[15]. Elsewhere, experimental infections of cattle with *Mycobacterium avium* subsp. *paratuberculosis* (MAP) have shown that a small proportion of the infected animals were misclassified as TB reactors when using the IFNγ test, but not with the SICTT [17].

In this study, we investigated the risk factors associated with IFNγ false positive tests in cohorts of animals in herds initially selected with no recent history of *M. bovis* infection in the herd. The risk factors considered in relation to whether or not an animal tested false positive in the IFNγ test were: age at the time of the index test, geographic region, month that the animal was tested at the index test, breed, herd enterprise type, sex and animal class.

## Material and methods

The blood samples on which the tests were carried out were diagnostic samples taken as part of the national TB eradication programme, which is subject to the EU trade Directive 64/432/EEC, which governs nature and frequency of testing. This is stated at the end of the Materials & Methods, Study population. The results presented in this study are a computer analysis of national databases containing the test results on the national herd. The data on particular animals involved in the study were filtered from the main databases using the criteria described. Details of the selection of these herds is published elsewhere [11].

### Study population

The population of cattle recruited to the study were from Irish herds identified as having a very low infection risk [11]. Details of herd selection are provided elsewhere [11], but relate to an absence of evidence of TB during both field and abattoir surveillance both on the farm and in the broader locality during the previous 5 years. At their annual herd SICTT test (index test) between April and December 2008, all animals greater than 6 months of age at day one (the day of tuberculin injection) of the herd test were sampled and included in the study. Blood was collected from each animal on the same day as the skin test but prior to injection with tuberculin. Testing of animals was conducted according to the EU trade Directive 64/432/EEC.

### Single intradermal comparative tuberculin test (SICTT)

The SICTT was carried out by intradermal injection of cattle with 0.1 mL PPD-bovine (1 mg/mL, 30 000 IU, ID Lelystad) and 0.1 mL PPD-avian (0.5 mg/mL, ID Lelystad, 25 000 IU) at sites 12 cm apart in the mid-neck region using a McLintock tuberculin syringe. Skin thicknesses were measured in mm at both sites before the intradermal injection and after 72 h. Two interpretations of the skin test were used in this study: “standard interpretation” (SICTT[standard]) if the bovine reaction was both positive (≥ 4 mm) and exceeded the avian reaction by more than 4 mm, and “severe interpretation” (SICTT.[severe]) if the bovine reaction was either positive (≥ 4 mm) or inconclusive (> 2 mm) and exceeded the avian reaction.

### Production and measurement of IFNγ

This test was conducted as previously described [11,13]. Up to 10 mL of heparinised blood were collected from each animal and 1.5 mL aliquots were dispensed into individual wells of 24-well tissue culture plates Costar, UK) containing either PPD-bovine (ID Lelystad), PPD-avian (both at 20 ug/mL) or PBS as a non-stimulating control. The cultures were incubated for 16 h at 37 °C in a humidified atmosphere with 5% CO2 before harvesting of plasma supernatants by centrifugation. Prior to assay, samples were stored at +4 °C where appropriate. IFNγ production was measured in duplicate samples by sandwich ELISA [12] using a commercial diagnostic kit (Bovigam, CSL Limited, Parkville, Australia). Absorbance values at 450 nm were converted to OD units using the formula, OD450 × 1000. A sample was considered positive when the OD450 of the PPD-bovine stimulated sample exceeded 100 OD units, was greater than the nil un-stimulated sample by 50 OD units and was greater than the PPD avian stimulated sample. The formula to define the test result for each animal is optimized for sensitivity with respect to lesion detection and positive *M. bovis* culture, and is currently adopted as the official interpretation in the Irish bovine TB eradication program [18].

### Follow up of animals

The *M. bovis* exposure status of study animals was followed from their initial index test (SICTT and IFNγ) in 2008 until either the end of the study, in December 2010, or until slaughter, whichever came first. Thus, all animals were in the study for a maximum of 32 months. For each animal, the exposure status was determined from either SICTT or post-mortem (PM) results either at the index test or in the time period following the index test (i.e., at the following annual SICTT). Animals were categorised using the standard or severe interpretation of the SICTT at the index test or in the follow-up period (note, if an animal was positive to the standard interpretation then it would also be positive to the severe interpretation and was, therefore, classified as SICTT[standard] positive). The categories used are described in Table 1.

**Table 1:** Future (to end of 2010) infection status of all animals by test result at time of the index test (2008).

**Future disease status**	**Gamma-interferon result**		
	**Neg**	**Pos**	**Total**
Negative	**1958**	**199**	**2157**
SICTT[Severe] + ve at index test			0
SICTT[Severe] + ve after index test	12	8	20
SICTT[Standard] + ve at index test		10	10
SICTT[Standard] + ve at index test/PM + ve	1	1	2
SICTT[Standard] + ve after index test	2	1	3
SICTT[Standard] + ve after index test/PM + ve	3		3
PM + ve after index test	2		2
Total	1978	219	2197
% positive	1.0	9.1	

### Study population: gamma-interferon false positives

All animals that tested negative to the SICTT at the index test and in the follow-up period, and also negative at post-mortem were considered further. Of these, animals that tested positive to the IFNγ at the index test were assumed to be “false positive” to the IFNγ test. This subset of all “negative” animals was used to estimate the specificity of IFNγ based on the assumption that these animals were ‘true negatives’. In addition these animals were considered further to identify risk factors associated with false positive reactions to the IFNγ test. The risk factors considered in relation to whether an animal gave a “false positive” reaction or not were: age at the time of the index test (note for animals born prior to 1996 the date of birth was not recorded, therefore all animals with a missing date of birth were assigned to age 13); geographic region; month that the index test was carried out; breed (cross-bred animals were assigned to the predominant breed); herd type (beef, dairy, suckler and mixed); sex and animal class (bull, steer, cow and heifer).

### Univariable data analysis

The proportion of animals that were positive to the IFNγ test was compared for each of the risk factors. Each risk factor was tested separately using the model described below.

### Multivariable data analysis

A logistic generalised estimating-equation (GEE) model was developed, the outcome variable being whether an animal tested positive to the IFNγ test at the index test. A compound-symmetry correlation was used to account for the correlation between animals within the same herd. All the risk factors were treated as categorical variables within the model. The age variable was categorised based on quartiles of the age distribution. The choice of whether to include either animal class or age and gender in the full model was assessed by comparing the QIC (Quasilikelihood under the Independence model Criterion) statistic from the univariable models. A backward-selection procedure was used to eliminate terms from the model based on a generalised score test (*p* > 0.05). Consistent estimates of coefficient standard errors were obtained using the empirical covariance matrix of parameter estimates resulting from the GEE method. The models were fitted using the SAS GENMOD procedure. An assessment of the goodness-of-fit was obtained by examining residuals. The Pearson residuals were examined using an index plot and a half normal plot with simulated envelope [19].

An Alternating Logistic Regression (ALR) model was also fit to the data in order interpret the measure of association within herds. For ALR models, within herd correlation was measured in terms of odds-ratios for 2 animals within the same herd [20].

## Results

### Study population

There were 2197 animals in 136 herds selected from the very low risk areas tested with SICTT and the IFNγ at the index test. The primary enterprise type in these herds was either suckler (91 herds), beef (29 herds), dairy (15 herds), or other (1 herd).

Of these animals, 1978 tested negative and 219 tested positive to IFNγ (Table 1). Among the animals that tested negative to IFNγ, 8 (0.4%) were either positive to the SICTT [standard] or positive on post-mortem examination, and a further 12 (0.6%) were positive to the SICTT [severe] by the end of the study period. There was slight agreement (Kappa = 0.086, 95% CI: 0.038 to 0.134) between the SICTT [Standard] and the IFNγ at the index test. Among the 219 IFNγ positive animals 12 (5.5%) were positive to the SICTT [standard] or at post-mortem, and a further 8 (3.6%) were positive to the SICTT [severe]. The proportion of animals that were positive to either the SICTT or positive at the PM at the index test or in the follow-up period was significantly higher amongst the IFNγ positive animals compared with the IFNγ negative animals, (*p* < 0.001). The odds of being positive at the index test or in the follow-up period were 9.8 times (95% CI: 5.2 to 18.6) higher for the IFNγ positive animals compared with the IFNγ negative animals.

### Gamma-interferon false positives

There were 2157 study animals that were negative to both the SICTT and at PM. Of these, 199 were positive to the IFNγ test. Based on this subset of animals, the estimated specificity of the IFNγ test was 90.77% (95% CI: 89.46% - 91.94%). By the end of the study period 49% of the animals had been slaughtered. There was no significant difference in the proportion slaughtered by IFNγ status (*p* = 0.910). These animals were found in 136 herds, of which 60 had one or more IFNγ positive animal(s) (ranging from 1 to 26 positive animals per herd, median = 2). Of these herds 36 had more than one IFNγ positive animal and 14 had 5 or more. Of these 14 latter herds there was a significant difference by region (*p* < 0.001), with 9 of them located in the South West and 8 of those herds located in Co. Limerick. Of the 4 herds with 10 or more IFNγ positives, 3 were located in Co. Limerick.

### Univariable analysis

The number and proportion of animals that were positive to the IFNγ test, categorized by each of the risk factors is shown in Table 2. Region was found to be a significant risk factor (*p* = 0.029) with the highest proportion of false positive IFNγ reactors (18.4%) detected in the south-west (SW) of Ireland, while the lowest proportion (5.0%) was detected in south-east counties (SE). The month when the testing was carried out was also a significant risk factor (*p* = 0.043) with October to November being the highest risk period for disclosing false positive IFNγ reactors (13.7-15.2%) and testing in July posing the lowest risk (4.8%). The age of the animal at the index test also appeared to be a borderline significant risk factor (*p* = 0.059), with a higher proportion of animals aged > 5 years (11.1%) testing IFNγ positive compared with animals aged > 6 months – 2 years old (8.0-8.1%). Other measured variables, including animal breed, herd enterprise, gender and animal class, showed no significant differences in disclosing a false-positive IFNγ reactor.

**Table 2:** Univariate analysis: proportion of animals positive to the IFNγ test at the index test, by risk factors.

**Variable**	**Category**	**No. of animals**	**No. positive to gamma interferon**	**% positive to gamma interferon**	***p*-value**
Age (years)	0.5–1	807	65	8.1	0.059
	2	351	28	8.0	
	3–4	334	32	9.6	
	≥ 5	665	74	11.1	
Region	NE	204	17	8.3	0.029
	NW	995	59	5.9	
	SE	398	20	5.0	
	SW	560	103	18.4	
Month	4	185	10	5.4	0.043
	5	212	13	6.1	
	6	266	26	9.8	
	7	273	13	4.8	
	8	319	31	9.7	
	9	273	15	5.5	
	10	479	73	15.2	
	11	102	14	13.7	
	12	48	4	8.3	
Breed^1^	AA	280	21	7.5	0.408
	BB	88	7	8.0	
	CH	497	38	7.6	
	FR	412	62	15.0	
	HE	130	17	13.1	
	LM	404	31	7.7	
	SH	54	2	3.7	
	SI	198	11	5.6	
	Other	94	10	10.6	
Herd type	Beef	307	35	11.4	0.072
	Dairy	569	85	14.9	
	Mixed	20	1	5.0	
	Suckler	1261	78	6.2	
Gender	Female	1671	170	10.2	0.108
	Male	486	29	6.0	
Animal class^2^	Bull	38	3	7.9	0.206
	Cow	1119	116	10.4	
	Heifer	550	54	9.8	
	Steer	448	26	5.8	

^1^ Animal Breed: AA (Aberdeen Angus), BB (Belgian Blue), CH (Charolais), FR (Friesian), HE (Hereford), LM (Limousin), SH (Shorthorn), SI (Simmental). Regions: NE (North-East), NW (North-West), SE (South-East), SW (South-West).

^2^ Two animals had no animal class recorded and 1 pregnant heifer was recoded to heifer due to small numbers.

### Multivariable analysis

The initial multivariable logistic GEE model included all of the measured variables where *p* < 0.2 at the univariable level, and in the final model only age and region were found to be significant risk factors (Table 3). Animals aged 5 or more had 1.4 times the odds of being a false positive IFNγ reactor compared to animals aged 6 months -1 year. In addition, the odds of a false positive IFNγ reactor were 2.2 higher in the SW region compared to the NE. The intra-herd correlation from fitting a compound symmetry correlation was 0.068. The ALR model gave similar results to the GEE model (Table 3), however, this measure of within herd correlation provided a more meaningful interpretation. From the ALR model the log odds of the within herd correlation was 0.592 (*p* < 0.001). This can be interpreted as the odds of finding an IFNγ false-positive animal in a herd was 1.81 times higher when it was known that another animal in the same herd was also positive.

**Table 3:** **Logistic Generalised Estimating-Equation** (**GEE) and Alternating Logistic Regression (ALR) model of the probability of a false positive result to the IFNγ test.**

**Variable**	**Label**	**b**	**S.E.**	**OR**	**Low**	**Upper**	***p*-value**^**1**^	***p*-value**^**2**^
***GEE model***								
Constant		-2.55	0.41					
Age (years)	0.5-1	Referent						0.046
	2	-0.20	0.25	0.82	0.50	1.34	0.431	
	3-4	0.17	0.26	1.19	0.71	1.98	0.518	
	≥ 5	0.33	0.19	1.40	0.96	2.03	0.080	
Region^3^	NE	Referent						0.035
	NW	-0.36	0.43	0.70	0.30	1.62	0.400	
	SE	-0.26	0.56	0.77	0.26	2.30	0.643	
	SW	0.80	0.41	2.23	1.00	4.98	0.051	
*Ρ*		0.068						
***ALR model***								
Constant		-2.53	0.40					
Age (years)	0.5-1	Referent						0.045
	2	-0.19	0.25	0.83	0.51	1.35	0.445	
	3-4	0.16	0.27	1.18	0.70	1.99	0.542	
	≥ 5	0.34	0.20	1.40	0.95	2.06	0.088	
Region^3^	NE	Referent						0.026
	NW	-0.40	0.42	0.67	0.29	1.53	0.343	
	SE	-0.34	0.51	0.71	0.26	1.95	0.511	
	SW	0.76	0.40	2.13	0.97	4.71	0.061	
logOR		0.592	0.155				<0.001	

*b*coefficient, *S.E* Standard Error, *OR* Odds ratio.

^1^Wald test. ^2^ Generalised score test. ^3^ NE (North-East), NW (North-West), SE (South-East), SW (South-West).

## Discussion

In Ireland, a full-herd SICTT is conducted annually on all cattle herds as part of the TB eradication programme [18]. Although recognized as an imperfect test at an animal level, its performance characteristics at herd level provides sufficient sensitivity and specificity for use as a disease surveillance test to detect infected herds. The IFNγ assay is used as a supplementary test in conjunction with the SICTT in severely infected herds or in groups of animals where the reduced specificity is considered acceptable. The test is targeted at herds with a high probability of containing infected animals or at those herds chronically infected over a number of years. The assay conditions and test interpretation have been evaluated in Irish cattle so as to take account of the effects of infection with, or exposure to, other mycobacterial species and related micro-organisms sharing epitopes with *M. bovis *[13]. The relatively low specificity of the IFNγ test precludes its usage as a surveillance test because of the risk of disclosing false positive reactors. The estimate of specificity from the current study of 90.77% is similar to that estimated using latent class analysis without a gold standard, which estimated the specificity at 86.8% to 89.4% [11]. However, it is lower than from other studies such as Gormley et al. [13]. The earlier study was conducted on a small number of herds (*n* = 26) clustered in one county in the south of the country, and all were sampled within a narrow time-frame. In contrast, the current study used a much larger number of herds geographically spread across the country and therefore may better reflect the range of risk factors encountered during herd surveillance at national level.

This study was conducted in a very low risk TB population, which has helped in identifying some of the risk factors associated with disclosure of these IFNγ false positives. With respect to misclassification of the initial disease status of our study herds, there is always a level of uncertainty about the TB-free status of a herd due to the imperfect nature of the SICTT. With this low-prevalence herd population, these were defined based on a previous five-year history of non-disclosure of standard reactors in both the herd and the immediate locality, while recognizing that herds may have become infected during the course of the study. Indeed, a proportion of the initial IFNγ + ve/SICTT –ve animals did become SICTT + ve during the follow-up period of study. It is likely that the IFNγ test measured these true positives, possibly at an early stage of infection. The odds ratio of being positive to the SICTT/PM for IFNγ positive animals compared to IFNγ negative animals (OR = 9.8) is consistent with that reported in previous studies in Ireland and in the UK [13,21].

Though there is evidence that co-infection with MAP can reduce the sensitivity of the IFNγ test for bovine tuberculosis [22] there is no strong evidence that natural infection with MAP affects the specificity of the IFNγ test [23]. However, in a study on cattle experimentally infected with MAP, a proportion of animals were misclassified as TB reactors using the IFNγ test at different time points post-infection, including one animal that was misclassified at 44% of the sampling time points [17]. No animals were misclassified by the SICTT. By comparison, and as part of the same study, cattle experimentally infected with *M. avium* subsp *avium* were correctly classified at all sampling time-points. These results clearly illustrated the cross reactivity between the bovine and avian tuberculin, and the potential for MAP infection to compromise the performance of the IFNγ test through reduction of the test specificity. However, in our current study we have no evidence that the herds in the low prevalence areas were infected with any other pathogenic mycobacteria.

Following the application of strict criteria for exclusion of animals suspected of being truly infected in the very low prevalence population, the risk factors associated with the remaining false-positive animals were investigated. The univariable model highlighted the potential importance of region, the time (month) of testing and the age of the animal, however, only region and age were significant in the multivariable logistic GEE model. It is likely that each of these factors may be related to the non-specific sensitization of cattle with various non-tuberculous mycobacteria. In Ireland, the SICTT test is used routinely in all animals as it has been shown that the application of a single intradermal test, using just bovine tuberculin, would result in 8-12% of animals in Ireland and the UK testing positive [24]. Saprophytic mycobacteria have been shown to cause transient non-specific reactions to the SICTT in Irish cattle [25] and an indigenously isolated species, *Mycobacterium hiberniae* wa*s* found capable of inducing non-specific reactions to tuberculin when administered parenterally or by the oral route [25]. In Ireland, the geographic distribution of the sensitizing saprophytes may be responsible for the regional differences in the proportion of false positive animals disclosed. From the descriptive analysis at herd-level, a high proportion of herds with multiple IFNγ positive animals were located in one county, with evidence from the ALR model of within-herd clustering, suggesting a localised source of sensitisation. Likewise, changes in the temporal abundance of the biologically relevant saprophytes may account for the proportion of false positives at different times of the year, as indicated in the univariate model.

The increased risk of a false positive result with age may simply reflect an age-accumulated risk. There is evidence to show that very young animals (< 6 months) can non-specifically produce high levels of IFNγ, due to the presence of a higher proportion of IFNγ producing natural killer (NK) cells in this age cohort [26]. It is for this reason that the IFNγ test is not routinely applied in very young animals and that animals younger than 6-months of age were not included in the study of Clegg et al. [11]. Our data suggests that beyond the 6 months of age threshold, the risk of non-specific IFNγ responses increases with age.

The precise underlying mechanisms responsible for false positive reactions are largely unknown, although variations in the types and potency of tuberculin used, and the criteria used for interpretation of the test results can directly affect sensitivity and specificity of the test [8,16]. In a recent study carried out in the UK bovine and avian tuberculin from different sources were compared with regard to their diagnostic performance in cattle experimentally and naturally infected with *Mycobacterium bovis*[14]. Significant differences were measured between the sources and concentrations of tuberculins used, highlighting a potential need for standardisation of PPDs used in the IFN-gamma assay. In a study conducted in Italy on TB free herds, the source of tuberculin also had an influence on the specificity of the test [16]. In addition, that study also showed that beef cattle had a higher risk of disclosing false positive reactors compared with dairy herds, and type of housing for animals also had an effect. However, there was no effect of animal age on the false positive rate. Unlike the analysis of the risk factors in Ireland described here, there was no reporting of multivariable analysis of the risk factors.

A range of methodological issues were considered during the design and analysis of this study. The study was not originally designed to specifically identify risk factors for false positive reactions, therefore, the data available on risk factors for the study are those routinely collected as part of the Irish bTB eradication programme. There are other risk factors such as environmental factors on the farm and surrounding area and the history of other diseases on the farm that have not been considered in this analysis and may be useful to consider in future studies. Further, farmers may have been tempted to treat the IFNγ positive animals differently. For this reason, results from the IFNγ were not fed back to the farmer or local veterinary practitioner. This is reflected in the two groups (IFNγ negative and positive animals) having the same bTB detection slaughter rates during the study. The commonly used approach to account for clustering within herds, using Generalised Estimating Equations, was initially carried out within the analysis. However, an additional modelling approach, using an Alternating Logistic Regression model, was also developed in order to produce a more readily interpretable measure of the correlation within herds. The results of the two models were consistent giving us added confidence in their findings.

The differences in the risk factors identified in the current study and the Italian study [16] highlight the need for evaluation of IFNγ test performance in the environment where it is being routinely used. As any particular environmental cause is likely to vary both spatially and temporally, caution should be taken when extrapolating specificity estimates from one cohort of animals to another in a different environment or over time. In order to establish the true specificity of a test, it should be determined in unbiased cohorts of animals i.e. after due consideration of those risk factors that are known to influence test specificity, otherwise the true test specificity may be over-estimated. To circumvent problems associated with false positive animals, defined mycobacterial antigens such as ESAT6 and CFP10 can be included in the test as stimulating antigen to increase test specificity [27-29] as these proteins are encoded by genes absent in almost all environmental mycobacteria [30]. In addition, the cut-off values and algorithms used to interpret the test result can be modified to change the interpretation of the test [2]. If it believed that external factors (e.g. environmental sensitization) are compromising the test performance then, for example, the cut-offs can be changed to optimize the test specificity. However, any changes to the test format needs to be validated against the appropriate cohort of animals in order to build confidence that the test performance is optimized for a particular environment.

## Competing interests

The authors declare that they have no competing interests.

## Authors’ contributions

EG, AD, MG, SM, TC conceived and designed the study. MD, EG, AD generated the raw data. EG, MD analysed raw data. EG, TC performed statistical analysis. EG, TC wrote paper with contributions from AD MG MD SM. All authors read and approved manuscript.
